# Fatal Complications in *Candida parapsilosis* Endocarditis—A Case Report

**DOI:** 10.3390/jof11110817

**Published:** 2025-11-18

**Authors:** Sebastian George Smadu, Simona Camelia Tetradov, Luminita Ene, Simin Aysel Florescu, Dragos Stefan Lazar

**Affiliations:** 1 Department of Infectious Diseases, “Carol Davila” University of Medicine and Pharmacy, 37 Dionisie Lupu Street, 020021 Bucharest, Romania; sebastian-george.smadu@drd.umfcd.ro (S.G.S.); simin.florescu@umfcd.ro (S.A.F.); dragos.lazar@umfcd.ro (D.S.L.); 2 ”Victor Babes” Clinical Hospital for Infectious and Tropical Diseases, 281 Mihai Bravu Street, 030303 Bucharest, Romania; luminita.ene@spitalulbabes.ro

**Keywords:** fungal endocarditis, prosthetic valve endocarditis, *Candida* endocarditis

## Abstract

Fungal endocarditis, despite being a rare diagnosis, has a higher morbidity and mortality rate compared to bacterial endocarditis. *Candida* species are the most common isolated pathogens involved in fungal endocarditis. Diagnosis is suspected in patients with underlying conditions such as cancer, myelodysplastic syndrome, diabetes, or intravascular catheters, where the modified Duke criteria apply. Management of the patient requires a multidisciplinary team (cardiologist, infectious diseases consultant, cardiac surgeon) along with antifungal treatment. We present the case of a 60-year-old male with biological prosthetic aortic valve replacement in the previous year for bicuspid aortic stenosis, admitted for a 5-day history of fever, nausea and minor urinary symptoms. The blood cultures were positive for *Candida parapsilosis*. Transthoracic cardiac ultrasound revealed a hypoechogenic mass attached to the aortic valve at the prosthetic fixation site. Although diagnosis was rapidly confirmed and treatment was administered shortly after first suspected, the patient developed, at first, cavernous sinus thrombosis and, later, fatal ST elevation myocardial infarction. The patient died despite efficient antifungal therapy, initially with Anidulafungin in monotherapy and later in combination with Fluconazole. The reported case emphasizes the importance of managing fungal endocarditis, the need for urgent diagnostic attention and multidisciplinary team approach by infectious diseases specialist, cardiologist, neurologist and heart surgeon.

## 1. Introduction

Prosthetic valve endocarditis (PVE) is the most severe form of infective endocarditis. In the early-onset form of PVE, *Staphylococcus aureus* is the most frequent isolate, although coagulase-negative *Staphylococci* and *Candida* species are also identified. In late-onset PVE, *Streptococcus* and *Staphylococcus* species are typically diagnosed [1].

In fungal endocarditis, *Candida* species are the most common isolated pathogens, while *Aspergillus* species and other fungi are isolated in a minority of cases [2,3].

Risk factors associated with invasive fungal infection are immunosuppression, neoplasia, myelodysplastic syndrome, surgery, diabetes, total parenteral nutrition, indwelling catheters, antibiotic use, and prosthetic valve replacements [4].

Prosthetic valve replacement represents a predisposing condition for *Candida* infective endocarditis, with biologic prostheses accounting for approximately 60% of the cases, occurring more often than mechanical valves [5].

Diagnosing fungal PVE is challenging given its rarity. Underlying health condition predisposing for invasive fungal infection and Modified Duke criteria are essential for diagnosis. Positive blood cultures are considered the “gold standard” in infective endocarditis [6,7].

Detecting fungemia through blood cultures is the recommended laboratory method for investigating and establishing the etiology. Routine blood cultures are incubated on automated continuous monitoring systems for up to five days, with no need for prolonged incubation or terminal subculture [2,8].

Identifying yeast from positive blood cultures can be achieved through various methods. Automated systems, such as Matrix-Assisted Laser Desorption/Ionization Time-of-Flight (MALDI-TOF), are becoming more common and easy to perform. After identification, antifungal susceptibility testing is performed on all positive cultures using broth microdilution or automated systems, with results interpreted based on the EUCAST Antifungal Clinical Breakpoints [9].

Other biomarkers such as 1-3-Beta-D-Glucan of Galactomannan Antigen are useful as an orientation tool toward fungal infection. 1-3-Beta-D-Glucan, component of the fungal wall, is detected in patients with invasive fungal infection with a sensitivity ranging from 76.7% to 100% [6].

Transthoracic and transesophageal echocardiography remains the first-line imaging technique in the diagnostic process. Transthoracic echocardiography is relatively inexpensive, widely available, and easy to perform, being sufficiently descriptive to characterize the valvular structure. The reported sensitivity for transthoracic echocardiography is 70% in native valve endocarditis and 50% in prosthetic valve endocarditis [7]. Positron Emission Tomography Computed Tomography (PET-CT) is an advanced imaging technique that characterizes valvular structures and captures metabolically active images in infectious endocarditis; however, its higher cost and radiation exposure limits its widespread use [6,7].

According to European Society of Cardiology Guidelines, fungal endocarditis is recommended to be managed by a combination of effective antifungal drugs and surgery, performed to control infection and remove the infected prosthetic valve [7].

Research has shown that surgery has not consistently been associated with better survival rates compared to medical management alone [5]. Antifungals are usually prescribed according to the species isolated in blood cultured and their sensitivity profiles. Amphotericin B is historically considered the first-line antifungal and had been used based on experience with invasive *Candida* infection. However, the newer echinocandins are a crucial part in management of fungal endocarditis due to their safety and tolerability profile compared to Amphotericin B, especially with a lower renal toxicity [10,11]. Recent data suggest that combined echinocandins with azoles therapy can improve survival compared to monotherapy [11].

Septic emboli are the most frequent complication of PVE, observed frequently in brain and spleen, although they can occur in many other organs. The prognosis remains poor despite efficient treatment, with a high mortality rate ranging between 30 and 80% [12,13,14].

## 2. Case Report

We present a case of a 60-year-old man who was hospitalized at “Doctor Victor Babes” Clinical Hospital for Infectious and Tropical Diseases-Bucharest Romania, on 24th March 2023, a tertiary hospital, dedicated to the diagnosis and treatment of infectious diseases, for a five-day history of fever (39.6 °C) and nausea.

Past medical history includes the following: bioprosthetic aortic valve replacement for bicuspid aortic stenosis one year prior, valvular cardiomyopathy with Left Ventricular Ejection Fraction of 35%, and recurrent urinary tract infections (UTI). The clinical examination on admission was unremarkable, apart from minor dysuria and increased urinary frequency. Giordano’s sign was negative, and no heart murmurs were observed. The performed blood tests revealed mild leukocytosis (13,000/mmc) with left shift formula (9300/mmc neutrophils) and increase in inflammatory response (ultrasensitive C Reactive Protein 17.6 mg/dL, Procalcitonin 2.95 mg/mL) and a mild renal impairment (Creatinine 1.6 mg/dL with an eGFR of 47/mL/min/1.73 m^2^).

At first, suspecting a UTI based on prior history and presenting symptoms, blood cultures and urinalysis were performed, and empiric antibiotic treatment was initiated with Piperacillin/Tazobactam.

After 36 h of incubation, blood cultures drawn on admission came back positive for oval-shaped cells suggestive of fungal infection. Consequently, two blood cultures were drawn, and intravenous Fluconazole was initiated at a 400 mg daily dose, on the 3rd day of hospitalization. All blood cultures were positive for yeast that the following days were identified as *Candida parapsilosis* by Matrix-Assisted Laser Desorption/Ionization Time-of-Flight (MALDI-TOF/BRUKER, Billerica, MA, USA) technique. Antifungal susceptibility testing was performed automatically by VITEK 2C (bioMérieux, Marcy-l’ Étoile, France), revealing sensitivity to all tested antifungals, with the following minimum inhibitory concentrations (MIC): Fluconazole < 0.5 mg/L, Voriconazole < 0.12 mg/L, Amphotericin B < 0.25 mg/L. An additional E test performed for Anidulafungin showed an MIC of 0.50 mg/L.

Part of routine protocol of screening for infective endocarditis in patients with predisposing heart condition, adding to the fact that the first blood culture was positive, on day 4, the patient was referred to the cardiological team that performed the aortic valve replacement 1 year prior for an evaluation and an echocardiography.

**Figure 1:**
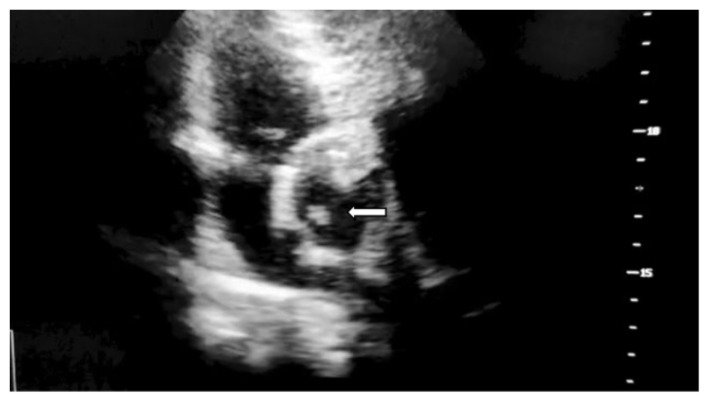
Transthoracic echocardiography hyperechogenic, serpentiform structure measuring 9.9 mm in maximum length, adherent to the aortic surface of the bioprosthetic valve at the fixation site with preserved mobility, marked with white arrow.

Transthoracic echocardiography performed on the 4th day confirmed a vegetation associated with the bioprosthetic valve sown in Figure 1. Slight prosthetic mismatch observed immediately after surgery persisted: maximum velocity at the aortic level of 3.5 m/s, velocity time integral of 60 cm, mean/maximum gradient of 24/49 mmHg across the prothesis, short acceleration time at the prothesis less than 100 mm, maximum velocity in the descending aorta of 0.8 m/s.

Ophthalmological examination performed also on the 4th day revealed lesions typical of Roth spots in the infratemporal region of the left eye.

The diagnosis of aortic prosthetic valve acute endocarditis with *Candida parapsilosis* was confirmed based on the modified Duke Criteria (Table 1).

The patient was referred to the surgical team, who initially treated the aortic stenosis with biological valve replacement and opted that the patient should continue antifungal therapy in the infectious diseases department, with the perspective to reintervene subsequently to address the endocarditis.

Considering that the patient’s eGFR of 47 mL/min/1.73 m^2^, was indicative for Stage 3a Chronic Kidney Disease, Amphotericin B was not alleged to be a viable treatment option due to its nephrotoxicity potential, in a patient with a preexisting renal condition. Therefore, on day 5 of admission, the patient’s antifungal regimen was switched from intravenous Fluconazole to Anidulafungin (200 mg on day 1 followed by 100 mg daily), and antibiotics were stopped, as urine culture and blood cultures did not identify any bacterial infection.

**Figure 2:**
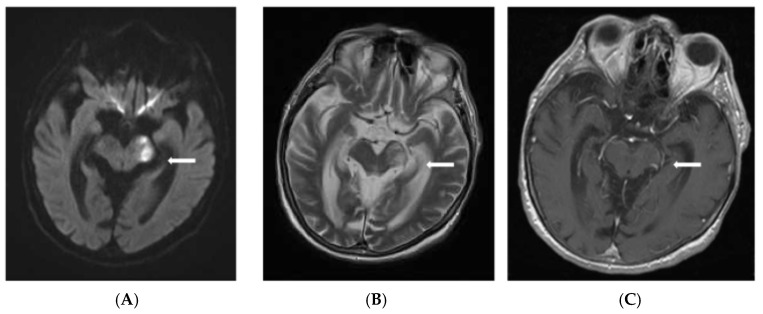
Lesional area located at the anterior portion (crus cerebri) of the left cerebral peduncle suggestive for acute ischemia, with water diffusion restriction (**A**) and high signal on T2-weighted MRI (**B**), hyposignal on T1-weighted Spin Echo MRI, without contrast enhancement with a slight increase in volume (**C**) marked with the white arrows.

**Figure 3:**
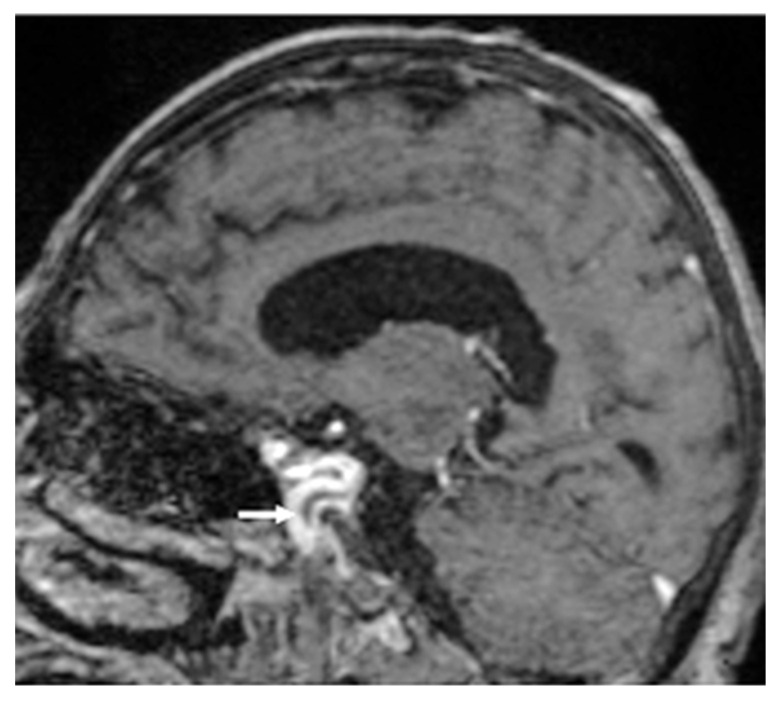
Sagittal T1-weighted Spin Echo Sequence in MRI showing heterogenous filling defect in the left cavernous sinus, suggestive for thrombosis marked with the white arrow.

On the 11th day of medical care, the patient became agitated with psychiatric signs and developed right sided hemiparesis, right oculomotor palsy, anisocoria, and non-reactive mydriasis. Based on the clinical examination, the neurologist suspected cavernous sinus thrombosis secondary to cardioembolic fungal stroke. The brain Magnetic Resonance Imaging (MRI) indicated both supratentorial and infratentorial multiple brain lesions, consistent with cerebral infarcts (Figure 2) and cavernous sinus thrombosis (Figure 3).

After the cerebral embolization episode, the patient presented reemergence of fever. As blood cultures were still positive for *Candida parapsilosis*, dual antifungal therapy was administered, including both intravenous Fluconazole and Anidulafungin. The patient was referred to the cardiologist for a new evaluation. The subsequent echocardiography, conducted two weeks after diagnosis, revealed the same cardiac parameters as the previous one, but the most important finding was the absence of the previously observed vegetation.

An interdisciplinary team comprising a cardiologist and a cardiac surgery specialist decided, after careful consideration, that valve replacement was inopportune at this time. The argument was that this was due to the stable cardiac function, unaltered valve function, and absence of the previously seen mass. The following days, neurologic motor deficits and fever persisted, the psychiatric symptoms worsened, although vital signs remained stable.

**Figure 4:**
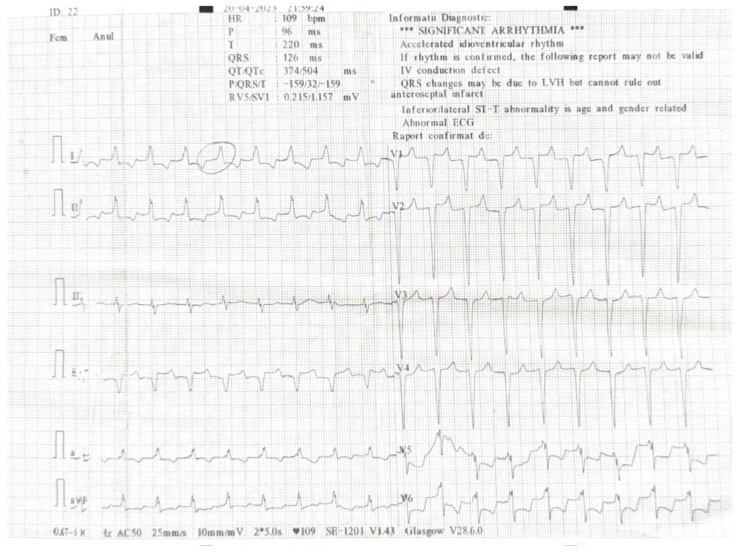
The 12-lead ECG performed at day 22 shows poorly visible atrial activity, most likely sinus rhythm, intraventricular left-sided conduction delay with mixed repolarization abnormalities secondary and ischemic. The positive Sgarbossa criteria were consistent with acute myocardial injury.

On the 22nd day following diagnosis, the patient clinical state suddenly worsened, marked by severe hypotension and unresponsiveness to fluid resuscitation, resulting in shock that required immediate administration of vasopressor support with Noradrenaline. The patient’s vital signs monitoring showed repolarization abnormalities on cardiac leads. The 12-lead Electrocardiogram (ECG) performed was suggestive of myocardial ischemia (Figure 4). The on-call physician highlighted the abnormal QRS complex, as shown in the image. Troponin levels doubled in value within a one hour period, on seriate determination, peaking at 719 ng/L.

Initial interventions were focused primarily on patient stabilization, in close collaboration with the intensive care specialist. Despite the intensive care procedures, the patient’s already critical state worsened, making the transfer to the cardiological center inadvisable due to transport-associated risks. The clinical condition gradually worsened, resulting in cardiorespiratory arrest that was unresponsive to resuscitation efforts on day 22 of hospitalization.

Blood cultures were performed serially throughout the patient’s four weeks of in-hospital stay for monitoring negative status. All blood cultures collected prior and during antifungal treatment, along with the last one drawn on the day preceding death, were consistently positive for *Candida parapsilosis*.

A definitive diagnosis for evaluating the true extent of the infection emboli by performing anatomopathological examination of the heart and brain was considered in this case. However, the family refused the request for the autopsy examination, thereby limiting the ability to confirm the exact scale of the events preceding the patient’s death.

## 3. Discussion

We have presented the case of a 60-year-old man diagnosed with *Candida parapsilosis* prosthetic aortic valve endocarditis. Despite 25 days of efficient treatment, he had persistent fungemia and subsequently developed neurological and fatal cardiac complications.

The misleading initial presentation, which suggested a UTI, made the diagnosis challenging. Adding to the diagnostic difficulty, the clinical examination was unremarkable, lacking heart murmurs or signs of cardiac failure, even though the initial blood culture was drawn in the context of preexisting prosthetic valve, being part of routine screening for endocarditis for patients with predisposing heart conditions.

Positive blood cultures confirmed the suspected endocarditis, revealing the presence of fungi. The patient had a small prosthetic mismatch after surgery that could have favored the yeast infection.

The source of *Candida parapsilosis* infection remains uncertain, as the patient had no central venous catheter, no history of parenteral nutrition, and no recent hospitalization. We can hypothesize that the gastrointestinal translocation or bloodstream invasion from other colonization sites might have contributed to the infection. Antifungal therapy was initiated promptly after the diagnosis; therefore, delayed treatment is unlikely to have contributed to the poor outcome. The presence of an early postoperative perivalvular leak may have acted as an additional predisposing factor for Candida adherence and biofilm persistence at the prothesis tissue interface, consistent with the observation of Alexis et al., which may further explain the persistence of infection despite adequate antifungal therapy [15].

The pathogenic mechanism by which fungi adhere to the valvular endothelium through fungemia is not well understood; however, in animal models, it appears that the fibrin-rich complex resulting from damaged endothelium facilitates the adherence of the yeast [6].

Also, biofilm formation, a characteristic of *Candida* species, can contribute to persistence of the microorganism and invasiveness, even months after the valve replacement surgery [16].

Although transesophageal echography has a higher sensitivity than transthoracic echography in both native and prosthetic valves, transesophageal echography shows 96% sensitivity in native valves and 92% in prosthetic valves [7].

In our patient, the screening performed with transthoracic echography was enough for diagnosis and cardiac characterization.

*Candida* species contribute to approximately 2% of infective endocarditis etiologies, and out of all the *Candida* species, *Candida parapsilosis* appears in 6–41% of cases [1,5,17].

Amphotericin B is still considered the gold standard of antifungal therapy, being the first line of treatment. The preexisting renal impairment in this case was a key factor in opting for Anidulafungin. Recent research suggests that the Amphotericin B-based regimen versus echinocandins shows no significant difference in both in-hospital and one-year follow-up mortality [10]. Our decision in choosing Anidulafungin as first-line treatment, was supported by the randomized controlled trial by Reboli et al., which included patients with invasive fungal infections, mostly candidemia, where Anidulafungin showed a higher overall treatment success at the end of intravenous therapy compared with Fluconazole (75.6% vs. 60.2%), with a trend toward lower mortality (23% vs. 31%) [18]. Other studies also support this approach, reporting higher success rates for Anidulafungin compared to Fluconazole (76% vs. 60%), recommending echinocandins as first-line therapy in severe invasive candidiasis [19]. Similarly, Ruhnke et al. reported favorable outcomes among critically ill patients diagnosed with invasive candidiasis treated with Anidulafungin, showing a treatment success rate of 69.5% and 90-day survival of 53.8% [20]. Although *Candida* endocarditis is rarely represented in clinical studies, the existing evidence supporting echinocandin use in critically ill patients is of great value and provides useful guidance in managing severe infections.

Septic embolization is the most frequent complication of infective endocarditis, primarily affecting the brain and spleen. The patient developed neurological complication with supratentorial and infratentorial ischemia and, afterwards, cavernous sinus thrombosis, which occurred probably due to the migration of the vegetation described on echocardiography.

Interestingly, the second cardiac complication occurred after transthoracic echography revealed the absence of vegetation, but the patient continued to be febrile with persistent fungemia. In this situation, transesophageal echocardiography could have been better suited for the visualization of millimetric persistent vegetations overlooked by transthoracic evaluation. The vegetation’s proximity to the coronary ostium significantly elevated the risk of coronary embolism. Comparing our patient with similar case reports of Candida parapsilosis endocarditis, its unfavorable outcome is consistent with the high mortality described in the literature, ranging between 35 to 60%, despite antifungal therapy [10,11,13,14,17]. Prosthetic valve involvement and persistent fungemia have consistently been associated with poor outcomes, despite efficient antifungal therapy. Survival rates tend to improve when surgical intervention is performed, in addition to antifungal therapy. The 2023 European Society of Cardiology Guidelines for management of endocarditis also emphasize that early surgical management in fungal endocarditis is strongly recommended, as delayed intervention can be associated with poor outcome, as prompt removal of infected prosthetic material is essential in achieving source control [7]. As most available data are based on a limited number of cases and not on cohort studies, the understanding of prognostic factors and optimal management relies on retrospective observation rather than systematic analysis. However, in our case, surgery was deferred and treatment relied on antifungals alone, which failed to achieve fungal clearance, resulting in persistent fungemia, clinical complications, and ultimately death. Treatment failure, despite in vitro susceptibility, can be explained in this case by several mechanisms. The first is biofilm formation, a characteristic feature of Candida species, which contributes to the persistence and invasiveness of the microorganism. Candida parapsilosis can form robust biofilms on prosthetic material, significantly reducing antifungal penetration and activity [16,21,22]. Another mechanism involved in treatment failure is the lack of surgical removal of the infected prosthesis. Although surgical intervention is generally considered to improve outcomes, reports in the literature show variable results regarding its impact on mortality [23].

As previously mentioned, the prognosis for such cases remains poor, with mortality rates often exceeding 50% [10].

## 4. Conclusions

The uncharacteristic presentation of this case of *Candida parapsilosis* prosthetic aortic valve endocarditis highlights the necessity of a high index of suspicion for endocarditis in patients with prior valve replacement.

As the patient had persistent fungemia, initial combined antifungal therapy might have worked similarly on other severe fungal infections in preventing complication. Surgical valve replacement might had changed the outcome, although data suggest that the mortality rates remain unchanged regardless of surgical cure.

A multidisciplinary team comprising an infectious disease specialist, cardiologist, neurologist, and cardiac surgeons, along with other specialists, is crucial for the complex management of these patients.

## Figures and Tables

**Table 1:** Modified Duke Criteria positive fulfilled.

Major Criteria	Minor Criteria
Three positive blood cultures for *Candida parapsilosis*	Roth spots-immunologic phenomena
9.9 mm vegetation present on the prosthetic aortic valve	Fever-documented
	Predisposing heart condition: prosthetic aortic valve 1 year prior

## Data Availability

The original data presented in the case report are included in the article, further inquiries can be directed to the corresponding author.

## Abbreviations

The following abbreviations are used in this manuscript:

PVE	Prosthetic valve endocarditis
MALDI-TOF	Matrix-Assisted Laser Desorption/Ionization Time-of-Flight
EUCAST	European Committee of Antimicrobial Susceptibility Testing
PET-CT	Positron Emission Tomography Computed Tomography
UTI	Urinary tract infections
CRP	C Reactive Protein
eGFR	Estimated Glomerular Filtration Rate
MIC	Minimum inhibitory concentrations
MRI	Magnetic Resonance Imaging
ECG	Electrocardiogram
